# Accumulation of 5-hydroxynorvaline in maize (*Zea mays*) leaves is induced by insect feeding and abiotic stress

**DOI:** 10.1093/jxb/eru385

**Published:** 2014-09-30

**Authors:** Jian Yan, Alexander E. Lipka, Eric A. Schmelz, Edward S. Buckler, Georg Jander

**Affiliations:** ^1^Boyce Thompson Institute for Plant Research, Ithaca, NY, USA; ^2^Institute for Genomic Diversity, Cornell University, Ithaca, NY, USA; ^3^Center for Medical, Agricultural, and Veterinary Entomology, United States Department of Agriculture, Agricultural Research Service, Chemistry Research Unit, Gainesville, FL, USA; ^4^United States Department of Agriculture – Agricultural Research Service, Robert W. Holley Center for Agriculture and Health, Ithaca, NY, USA; ^5^Department of Plant Breeding and Genetics Cornell University, Ithaca, NY, USA

**Keywords:** Aphid, drought, 5-hydroxynorvaline, maize, *Rhopalosiphum maidis*.

## Abstract

Plants produce a wide variety of defensive metabolites to protect themselves against herbivores and pathogens. Non-protein amino acids, which are present in many plant species, can have a defensive function through their mis-incorporation during protein synthesis and/or inhibition of biosynthetic pathways in primary metabolism. 5-Hydroxynorvaline was identified in a targeted search for previously unknown non-protein amino acids in the leaves of maize (*Zea mays*) inbred line B73. Accumulation of this compound increases during herbivory by aphids (*Rhopalosiphum maidis*, corn leaf aphid) and caterpillars (*Spodoptera exigua*, beet armyworm), as well as in response to treatment with the plant signalling molecules methyl jasmonate, salicylic acid and abscisic acid. In contrast, ethylene signalling reduced 5-hydroxynorvaline abundance. Drought stress induced 5-hydroxynorvaline accumulation to a higher level than insect feeding or treatment with defence signalling molecules. In field-grown plants, the 5-hydroxynorvaline concentration was highest in above-ground vegetative tissue, but it was also detectable in roots and dry seeds. When 5-hydroxynorvaline was added to aphid artificial diet at concentrations similar to those found in maize leaves and stems, *R. maidis* reproduction was reduced, indicating that this maize metabolite may have a defensive function. Among 27 tested maize inbred lines there was a greater than 10-fold range in the accumulation of foliar 5-hydroxynorvaline. Genetic mapping populations derived from a subset of these inbred lines were used to map quantitative trait loci for 5-hydroxynorvaline accumulation to maize chromosomes 5 and 7.

## Summary

Short statement: 5-Hydroxynorvaline is an abundant, stress-induced non-protein amino acid in maize. At concentrations similar to those in maize leaves, 5-hydroxynorvaline inhibits *Rhopalosiphum maidis* (corn leaf aphid) reproduction on artificial diet.

## Introduction

Plants in their natural environment are subject to attack by numerous herbivorous insects. Nevertheless, due to a variety of physical and chemical defences, any individual plant is resistant to feeding by most species of insect herbivores. Chemical defences, in particular, are exceedingly diverse and play a key role in limiting the host ranges of insect herbivores (Fraenkel, 1959), either through direct toxicity (antibiosis) or by making plant tissue so unpalatable that the herbivores are encouraged to feed elsewhere (antixenosis).

Untargeted metabolite profiling of a typical plant leaf can show several thousand distinct compounds. A few hundred of these are known constituents of primary metabolism, such as sugars, amino acids and lipids, which are found in most or all plants. Additionally, in many well-studied plant species, structures of the more abundant secondary metabolites, which provide defence against herbivores and pathogens, have been confirmed. However, in any given plant species the vast majority of the constituent metabolites remain unidentified. Although there are undoubtedly unknown intermediates in primary metabolism, it is likely that many of the as-yet-unidentified plant metabolites contribute to defence against herbivores and pathogens.

Given the plethora of plant metabolites that can be detected by metabolite profiling, it can be difficult to target further research towards those that are most likely to be important for plant defence. However, a common property of defence-related plant secondary metabolites is that their production is induced in response to insect feeding. Many of these induced insect defence responses require the jasmonate-dependent signalling pathway. During plant defence elicitation, jasmonate is conjugated to isoleucine, forming a jasmonate–isoleucine conjugate that promotes ubiquitination of JAZ repressor proteins by SCF^COI1^, thereby triggering their degradation and promoting expression of early response genes (Howe and Jander, 2008). Methyl jasmonate, a more volatile compound, is readily converted to jasmonate by plants and triggers many of the same defence responses as jasmonate itself. Well-studied examples of plant secondary metabolites that are induced by insect feeding in a jasmonate-dependent manner include nicotine in tobacco (Dewey and Xie, 2013), glucosinolates in *Arabidopsis* (Schweizer *et al.*, 2013) and protease inhibitors in tomatoes (Howe *et al.*, 1996). Together, these observations suggest that the converse may also be true, that plant secondary metabolites with increased abundance in response to jasmonate treatment are likely to contribute to defence responses.

Maize (*Zea mays*), the world’s most productive grain crop, is fed upon by a large variety of insect herbivores (Meihls *et al.*, 2012). These insects have a diversity of feeding habits and consume all parts of the maize plant. For instance, beet armyworms (*Spodoptera exigua*) and larvae of several other lepidopteran species consume maize leaves, corn earworms (*Helicoverpa zea*) eat the seeds, European corn borers (*Ostrinia nubilalis*) make tunnels in the stalks, corn leaf aphids (*Rhopalosiphum maidis*) ingest phloem sap, corn rootworm larvae (*Diabrotica virgifera*) damage the root system and adult *D. virgifera* feed from the silks and pollen. Well-studied anti-herbivore defences in maize include small molecules such as benzoxazinoids (Frey *et al.*, 2009), chlorogenic acid (Cortés-Cruz *et al.*, 2003) and maysin (Rector *et al.*, 2003), as well as defence-related proteins such as protease inhibitors (Tamayo *et al.*, 2000), cysteine protease (Pechan *et al.*, 2000) and ribosome-inactivating proteins (Chuang *et al.*, 2014). As in the case of other tested plant species, many of the observed maize defence responses are regulated by jasmonate (Ankala *et al.*, 2009; Dafoe *et al.*, 2011; Christensen *et al.*, 2013).

As only a small fraction of the chemical makeup of maize leaves has been elucidated, it is likely that there are many as yet unknown defensive metabolites. Thus, a targeted search for metabolites that become more abundant after insect feeding and/or elicitation with plant signalling molecules would almost certainly identify novel chemical defences in maize. In particular, because non-protein amino acids have not been studied extensively in maize, these were made the focus of research to identify previously unknown defensive metabolites. High-performance liquid chromatography (HPLC) analysis of compounds that were more abundant after methyl jasmonate treatment identified 5-hydroxynorvaline. This non-protein amino acid has been reported in the seeds and/or leaves of legumes and grasses (Thompson *et al.*, 1964; Brown and Fowden, 1966; Carmo-Silva *et al.*, 2009; Rohlig *et al.*, 2009; Skogerson *et al.*, 2010; Du *et al.*, 2012), but very little research has been done to characterize its biosynthesis and function in plant metabolism. The production and accumulation of maize foliar 5-hydroxynorvaline in response to both biotic and abiotic stress are described here.

## Materials and methods

### Plants and growth conditions

Maize seedlings were grown in corn mix [produced by mixing 0.16 m^3^ Metro-Mix 360 (Scotts, Marysville, OH, USA), 0.45kg finely ground lime, 0.45kg Peters Unimix (Scotts), 68kg Turface MVP (Profile Products, Buffalo Grove, IL, USA), 23kg coarse quartz sand and 0.018 m^3^ pasteurized field soil] in 8×8cm plastic pots (≈200cm^3^), with 18 pots placed in each 36×53cm plant flat. Seeds were planted approximately 1.5cm deep and pots were placed in Conviron growth chambers (Conviron, Winnipeg, Canada) with a 16 h:8h light/dark photoperiod, 180 μmol photons m^−2^ s^−1^ light intensity, 23 °C and 60% humidity. For normal growth, plants were watered from below to maintain soil moisture. Unless otherwise noted, all plants were used for experiments when they were 2 weeks old, at the V2–V3 stage.

For assays of field-grown plants, B73 seeds were planted in a field in Aurora, New York, in May 2012. Plants were irrigated as needed, based on the weather conditions.

### Chemicals and solvents

[^13^C_5_^15^N_1_]Glu, [^13^C_5_^15^N_2_]Gln, [^15^N_2_]Gln, [^13^C_5_]Orn, [^13^C_6_]Arg and [^13^C_5_]Pro were purchased from Cambridge Isotope Laboratories (Tewksbury, MA, USA). Other chemicals and solvents were obtained from Sigma-Aldrich (St. Louis, MO, USA).

### Drought and cold stress

Prior to the drought experiment, each flat of 18 plants received approximately 600ml water each morning. Drought treatment was initiated by withholding water, whereas control plants continued to receive the same amount of water as previously. On each day of the experiment the second true leaf was harvested from individual plants for amino acid assays and the remainder of the harvested plants was discarded. For detached-leaf assays, the second true leaves were cut from B73 maize plants, weighed and left on paper towels in the same growth chamber in which the plants had been growing. After 0, 1, 2, 3, 4, 8, 16, 24, 32 and 48h, leaves were collected for amino acid assays. 5-Hydroxynorvaline content was calculated relative to the original fresh weight of each leaf to determine whether there is *de novo* synthesis as the leaves are drying. For cold stress experiments, B73 maize plants were placed at 4 °C in a cold room with a 16 h:8h light/dark photoperiod. Leaf tissue was collected at 7, 24, 48, 72, 96 and 120h after the start of the experiment. The second true leaf from each plant was harvested for 5-hydroxynorvaline assays.

### Treatment with plant growth hormones

For elicitation, the leaves of maize plants were sprayed with 0.45mM methyl jasmonate, 0.45mM salicylic acid, 0.45mM 1-aminocyclopropane-1-carboxylic acid or 0.1mM abscisic acid. Control plants were treated with water only. The second true leaf was harvested on the day of spraying (control) and on the consecutive 7 days thereafter. Leaves were frozen in liquid nitrogen and stored at −80 °C prior to amino acid assays.

### Insect rearing and plant infestation

B73 maize seedlings grown in growth chambers were used for *R. maidis* bioassays. Fifty adult aphids were confined on individual plants using microperforated polypropylene bags (15×3.6cm; PJP Marketplace, Philadelphia, PA, USA). Control plants were covered with the same bags. Leaf tissue was collected on days 1, 2, 3 and 4 after the addition of aphids, frozen in liquid nitrogen and stored at −80 °C for later analysis.

Artificial diet for *R. maidis* assays consisted of 440mM sucrose and 20 amino acids (Ala, 10mM; Arg, 16mM; Asn, 20mM; Asp, 10mM; Cys, 3.3mM; Glu, 10mM; Gln, 10mM; Gly, 10mM; His, 10mM; Ile, 6mM; Leu, 6mM; Lys, 10mM; Met, 5mM; Phe, 3mM; Pro,7mM; Ser, 10mM; Thr, 12mM; Trp, 4mM; Tyr, 2mM; Val, 7mM). 5-Hydroxynorvaline was added to the diet at 0, 0.01, 0.1, 0.5 and 5mM concentrations. Five wingless adult *R. maidis* were placed in a 30ml plastic cup that was covered with a Parafilm sachet containing 100 µl of the liquid diet. Aphid nymphs were counted after 4 days. For 5-hydroxynorvaline-uptake experiments, aphids were harvested for HPLC-based amino acid analysis after 4 days on the artificial diet.

For *S. exigua* experiments, eggs were obtained from Benzon Research (Carlisle, PA, USA). For plant experiments, neonate caterpillars were placed on 3 week-old B73 maize plants and covered with perforated plastic bags. On the third and seventh day after infestation, 100mg samples of leaf tissue were collected, both from the actual site of caterpillar feeding and from distal sites on leaves that had not yet been subjected to herbivory. Leaf samples were frozen at −80 °C prior to amino acid analysis. For artificial diet experiments, neonate *S. exigua* larvae were placed on artificial diet (Multiple Species Diet, Southland Products, Lake Village, AR, USA) supplemented with 0, 0.033 or 0.33mM 5-hydroxynorvaline. After 9 days the caterpillars were harvested and weighed.

### *Cochliobolus heterostrophus* infection

*C. heterostrophus* (southern corn leaf blight) was cultured as described previously (Huffaker *et al.*, 2011). Maize variety Golden Queen was inoculated using a previously described protocol (Schmelz *et al.*, 2011), with minor modifications. To inoculate young leaf tissue deep within the inner whorl, 100 µl of a 1×10^7^ spore ml^−1^ suspension was injected into the stem spanning a vertical 4cm region directly above the developing node tissues. This inoculum volume was divided between 12 equally spaced needle wound sites that extended through the diameter of the stem. Mock-treated plants were similarly damaged and treated with water. Inner whorl leaf tissues corresponding to the original treatment site were harvested after 4 days, when there were visible signs of infection, and frozen in liquid nitrogen.

### Collection of field samples

Tissue of 109 day-old B73 maize plants was collected from a field in Aurora, New York, in August 2012. At this time point the maize ears were still green and not senescing. Tissue samples (leaves, stems, underground roots, brace roots and tassels) were frozen in liquid nitrogen and stored at −80 °C for later analysis.

### Amino acid detection

Amino acids in maize leaf extracts were measured by HPLC with fluorescence detection. About 100mg of tissue from seedlings grown in growth chambers was weighed, frozen in liquid nitrogen in 2ml microcentrifuge tubes and ground to fine powder with 3mm steel balls using a Harbil model 5G-HD paint shaker (Fluid Management, Wheeling, IL, USA). Ground tissue was extracted with water (5 µl mg^−1^ of fresh tissue) containing 20mM HCl and 40 µM norleucine as an internal standard, the extracts were centrifuged at 3800 *g* for 20min at 23 °C, and the supernatants were saved for analysis. Tissue from field-grown plants and dry seeds from a previous harvest were pulverized in liquid nitrogen and extracted under the same conditions as tissue from seedlings grown in growth chambers.

For amino acid analysis, samples were derivatized using the AccQ-Fluor reagent kit (Waters, Milford, MA, USA) according to the manufacturer’s directions. Plant extracts (5 µl) were mixed with 35 µl of borate buffer and the reaction was initiated by the addition of 10 µl of 6-aminoquinolyl-*N*-hydroxysuccinimidyl carbamate reagent (AQC), followed by immediate mixing and incubation for 10min at 55 °C. Them 20 µl of each sample were injected into a Nova-Pak C_18_ column (3.9×150mm; Waters) and separated using a Waters 2790 HPLC pump system. Solvent A containing 10% AccQ-Tag Eluent A purchased from Waters; solvent B was acetonitrile/water (60:40). The gradient used for corn leaf analysis was: 0–0.5min, linear gradient from 0 to 3% B; 0.5–12min, linear gradient to 5% B; 12–15min, linear gradient to 8% B; 15–40min, linear gradient to 30% B; 40–42min, 30% B; 43–48min, 100% B; 49–55min, 100% A. The flow rate was 1.0ml min^−1^ and the column temperature was 37 °C. Eluted amino acid derivatives were detected using a Waters model 2475 fluorescence detector, with an excitation wavelength of 250nm and an emission wavelength of 395nm. Data were recorded and analysed using Waters Empower software.

For aphid amino acid analysis, aphids were collected from maize seedlings or artificial diet, weighed, frozen in liquid nitrogen, and ground to a fine powder. Powdered aphids were extracted in the same manner as maize leaves, using 100 µl buffer per mg of tissue. Five µl of extract were derivatized for amino acid analysis using the AccQ-Fluor reagent kit. Amino acid detection by HPLC was similar to that used for maize tissue: 0 to 3min, linear gradient to 6% B; 3 to 20min, linear gradient to 10 % B; 21 to 28min, 25 % B; 28 to 36min, linear gradient to 26 % B; 37 to 44min, 100 % B; 45 to 51min, 100% A. The flow rate was 1.0ml min^−1^ and the column temperature was 37 °C.

For nuclear magnetic resonance (NMR) structural determination, 1mg of the compound purified from maize tissue was dissolved in 100 µl of deuterated methanol (CD_3_OD) and analysed using an Inova 600 MHz NMR spectrometer (Varian, California, USA). The data were analysed by ^1^H, ^1^H-^1^H COSY NMR using MestreNova software. The NMR samples were also used for direct injection into a Waters/Micromass Quattro II triple quadrupole mass spectrometer with ESI detector. Waters MassLynx software was used for the data analysis.

### 5-Hydroxynorvaline purification

B73 maize plants were sprayed with 0.45mM methyl jasmonate four times over 2 days; about 1.6kg of fresh maize tissue were ground in liquid nitrogen and extracted with 8 l of water containing 20mM HCl. The extraction liquid was reduced to approximately 100ml by drying under vacuum. Crude extract (8ml) was mixed with 10ml of water. Aliquots (20 µl) of this solution were reacted with 20 µl of AQC and 140 µl of borate buffer at 55 °C for 10min. The reaction was dried under vacuum and then dissolved in 40 µl of water. Samples (20 µl) were injected into a Waters 2790 HPLC and separated as described above. The target peaks were collected by hand, samples from multiple HPLC runs were pooled and liquid was dried by lyophilization. Water was added to the dry product until it was completely dissolved. This liquid was loaded onto a Nova-Pak C_18_ HPLC column, the column was washed with water to remove salt, the target compound was eluted with 100% acetonitrile and the sample was dried under vacuum. This process was repeated until a total of approximately 1mg of the target compound was obtained.

### Synthesis of 5-hydroxynorvaline

In order to confirm the structure of the maize metabolite, authentic l *-*5-hydroxynorvaline was synthesized from l-glutamic acid as described previously (Garcia *et al.*, 2000). The reaction mixture was passed through a Dowex 50WX8-200 column, eluted with 80% acetonitrile and collected in tubes as 3ml fractions. From each fraction, 5 µl samples were derivatized using the AccQ-Fluor reagent kit and measured by HPLC fluorescence detection as described above. The fractions containing 5-hydroxynorvaline were combined and further purified using a Sephadex-LH-20 column, eluting with 100% methanol. The purity of the compound was confirmed by HPLC mass spectrometry.

### Stable isotope labelling and detection

Leaves detached from maize plants were inserted into 1.5ml microcentrifuge tubes containing 5mM labelled amino acids ([^13^C_5_^15^N]Glu, [^13^C_5_^15^N_2_]Gln, [^15^N_2_]Gln, [^13^C_5_] Orn, [^13^C_6_]Arg and [^13^C_5_]Pro) in water. Control leaves were treated with the corresponding unlabelled amino acids. The leaves were allowed to stand for 2h. After 2h, leaves were sprayed with 0.45mM methyl jasmonate. Tubes with leaf samples were placed under dome covers and allowed to stand for 2 days. Three leaves receiving the same treatment were combined as one replicate for derivatization and gas chromatography-mass spectrometry (GC-MS) analysis of amino acids.

Amino acid analysis by GC-MS was performed as described previously (Joshi and Jander, 2009), with minor modifications. Leaves were frozen in liquid nitrogen in 2ml tubes and ground to fine powder with 3mm steel balls using a Harbil model 5G-HD paint shaker. Ground tissue was taken up in 20mM HCl (500 µl per 100mg of fresh leaf tissue), the extracts were centrifuged at 3800 *g* for 20min at room temperature, and the supernatant leaf amino acid extracts (≈1.5ml) were applied to a Dowex-50 column and washed with 4ml of water. Samples were eluted with 6M NH_4_OH (2ml), the fractions were combined and the solutions were concentrated to 100–200 µl by lyophilization. These extracts were then completely dried under nitrogen flow at 70 °C. The residue was taken up in 50 µl *N*-methyl-*N*-(trimethylsilyl)trifluoroacetamide with 1% trimethylchlorosilane, the sample was heated for a further 1h at 100 °C and GC-MS analysis was performed using a Varian 1200L GC-MS (Agilent Technologies, La Jolla, CA, USA) with a DB-17ms capillary column. Spectra of known amino acids were assigned by reference to a spectral library of amino acid standards and the National Institute of Standards and Technology library.

### Genetic mapping and data analysis

Parental lines of the maize nested association mapping (NAM) population (Yu *et al.*, 2008; McMullen *et al.*, 2009; www.panzea.org) were planted in 3-fold replication, seedlings at the V2–V3 stage were sprayed with 0.45mM methyl jasmonate on two consecutive days and tissue from the second leaf of plants was harvested for amino acid analysis to determine which lines had a different 5-hydroxynorvaline content than the reference maize line, B73. Based on this assay, recombinant inbred lines derived from CML103, CML228, CML277 and Ky21 were planted in single replication, seedlings at the V2–V3 stage were sprayed with 0.45mM methyl jasmonate on two consecutive days and tissue was harvested for amino acid analysis.

A joint linkage procedure (Buckler *et al.*, 2009) was used to identify and locate common quantitative trait loci (QTL) across the four tested NAM families. Briefly, a joint stepwise model selection procedure was implemented to analyse 506 lines from the B73 × CML103, B73 × CML228, B73 × CML277 and B73 × Ky21 recombinant inbred line (RIL) populations, where the response variable was methyl jasmonate-induced 5-hydroxynorvaline abundance and 14772 markers distributed throughout the maize genome (RefGen v2, www.maizegdb.org) were considered for inclusion in the final model. After fitting a family main effect, marker effects nested within families were selected to enter or exit the model based on *P* values derived from a partial *F* tests calculated in TASSEL version 3.0 (Bradbury *et al.*, 2007). A permutation procedure (Churchill and Doerge, 1994) was conducted 1000 times to control the type I error rate at α = 0.05. The fiftieth smallest *P* value from these permutations, 2.17×10^−5^, was used as the threshold for each marker to enter the model. To circumvent the problem of moderately significant markers leaving the model after being selected, the exit *P* value was set at to two times the entry threshold (i.e. 4.34×10^−5^). The α = 0.05 support intervals for each QTL identified in the joint linkage analysis were calculated using a previously described procedure (Tian *et al.*, 2011). A series of *t* tests was conducted to assess the significance of the QTL effects within each family, controlling the false discovery rate at 5% (as in Hochberg and Benjamini, 1990). Finally, the phenotypic variance explained by each QTL identified in the joint linkage analysis was calculated (Li *et al.*, 2011).

Composite interval mapping of the RILs within each of the four tested NAM families was conducted using Windows QTL Cartographer (WinQTL) software version 2.5 (Wang *et al.*, 2012). Genetic marker data were downloaded from www.panzea.org. The WinQTL program settings were: CIM program module = Model 6: Standard Model; walking speed = 2 cM; control marker numbers = 5; window size = 10 cM; regression method = backward regression. The *P* < 0.05 LOD significance threshold was determined by running a permutation procedure (Churchill and Doerge, 1994) 500 times to control for the type I error at α = 0.05. Statistical tests were conducted using JPM Pro 10 software (www.JMP.com).

## Results

**Fig. 1.**
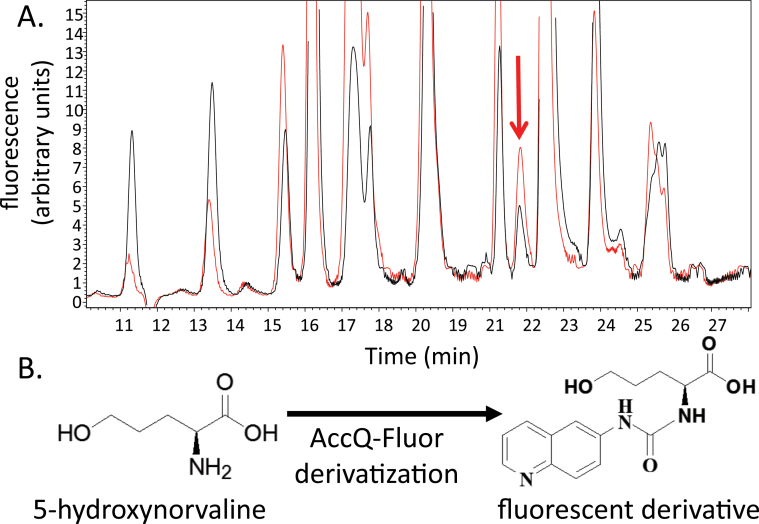
(A) HPLC-fluorescence detection chromatogram of maize amino acids. Black = uninduced; Red = induced with methyl jasmonate. The arrow indicates the induced peak of interest. (B) Structure of 5-hydroxynorvaline and the fluorescent derivative that is detected in A.

In a targeted search for non-protein amino acids that are induced by methyl jasmonate treatment, the free amino acid content in leaves of maize inbred line B73 was analysed by HPLC fluorescence detection after derivatization using the Waters AccQ-Fluor reagent kit. This assay identified an unknown amino acid peak that was more abundant in methyl jasmonate-treated plants than in controls (Fig. 1A). A combination of NMR and MS approaches was used to demonstrate that the unknown jasmonate-induced maize metabolite was 5-hydroxynorvaline (2-amino-5-hydroxypentanoic acid; Fig. 1B; Supplementary Fig. 1). In the ^1^H-NMR spectrum of the compound that was purified from the fluorescence derivatization assay described above, peaks were observed at δ_H_ 4.25 (t, 6.0 Hz), δ_H_ 1.76 (m) and 1.91(m), 1.64 (m) and δ_H_ 3.60 (t, 5.9 Hz). Correlation of δ_H_ 4.25 with δ_H_ 1.76 (m), δ_H_ 1.76 with δ_H_ 1.61 (m) and δ_H_ 1.61 (m) with δ_H_ 3.60 also were observed in the ^1^H-^1^H COSY 2D-NMR spectrum. MS spectra showed peaks with *m*/*z* 304 and 302 in the positive mass and negative ionization modes, respectively, indicating that the molecular weight of the fluorescence-derivatized compound was 303Da. As an *m*/*z* 171 daughter ion of the *m*/*z* 304 ion was obtained in the positive ionization mode, the molecular weight of the maize amino acid was determined to be 133Da (Supplementary Fig. 1). Together, these NMR and MS spectra showed that the identified maize metabolite was 5-hydroxynorvaline. Since 5-hydroxynorvaline is not commercially available, the authentic compound was synthesized from l-glutamate, as described previously (Garcia *et al.*, 2000; Supplementary Fig. 2). HPLC analysis showed that the synthetic compound was identical to the natural maize metabolite.

**Fig. 2.**
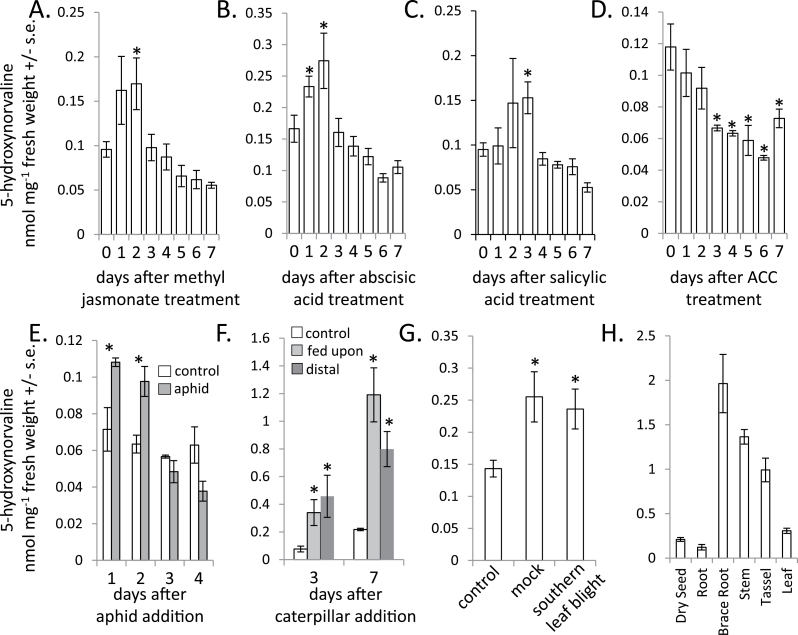
Induction and localization of 5-hydroxynorvaline in maize. Abundance of 5-hydroxynorvaline in maize seedlings after treatment with (A) 0.45mM methyl jasmonate, (B) 0.1mM abscisic acid, (C) 0.45mM salicylic acid or (D) 0.45mM 1-aminocyclopropane-1-carboxylic acid (ACC, an ethylene precursor). *N* = 3 for A–D; *P < 0.05, *t* test relative to day 0 sample. 5-Hydroxynorvaline accumulation in maize seedlings treated with (E) *R. maidis* (*N* = 3), (F) *S. exigua* (*N* = 3) and (G) mock-infected or infected with *C. heterostrophus* (southern leaf blight; *N* = 4). *P < 0.05 relative to controls, *t* test. (H) Abundance of 5-hydroxynorvaline in dry seeds and different parts of field-grown maize inbred line B73 (*N* =4).

In addition to methyl jasmonate (Fig. 2A), abscisic acid (Fig. 2B) and salicylic acid (Fig. 2C) treatment transiently increased 5-hydroxynorvaline accumulation in maize seedlings. In contrast, treatment with 1-aminocyclopropane-1-carboxylic acid (ACC, an ethylene metabolic precursor) reduced the 5-hydroxynorvaline abundance (Fig. 2D). Consistent with the observed induction by methyl jasmonate (Fig. 2A), 5-hydroxynorvaline accumulation was increased by aphid (*R. maidis*, Fig. 2E) and caterpillar (*S. exigua*, Fig. 2F) feeding. However, infection with a fungal pathogen, *C. heterstrophus*, did not increase 5-hydroxynorvaline concentrations to a higher level than mock-infected controls (Fig. 2G), despite the fact that *C. heterstrophus*-infected leaves showed visible tissue necrosis and mock-infected leaves did not.

Measurement of 5-hydroxynorvaline in different parts of mature B73 maize plants growing in the field showed that it is most abundant in above-ground vegetative tissue (Fig. 2H), suggesting that this metabolite could provide protection against herbivores like *R. maidis* and *S. exigua*. 5-Hydroxynorvaline concentrations in vegetative tissue of field-grown plants were considerable higher than those of seedlings in growth chambers. Smaller amounts of 5-hydroxynorvaline were detected in below-ground roots of field-grown plants. Dry seeds also contain 5-hydroxynorvaline, consistent with previous reports of small amounts of this amino acid in maize grain (Rohlig *et al.*, 2009; Skogerson *et al.*, 2010).

**Fig. 3.**
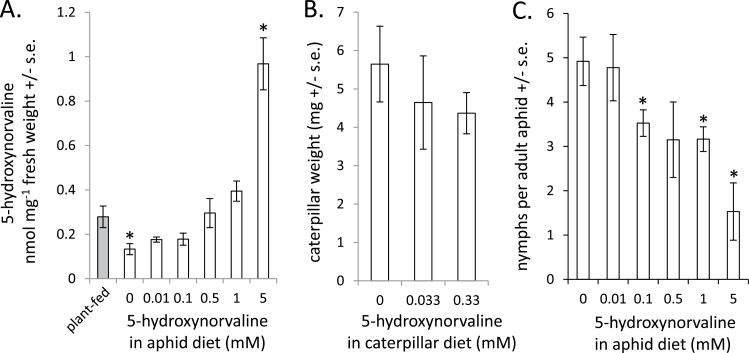
Effects of 5-hydroxynorvaline on *R. maidis* and *S. exigua*. (A) Concentration of 5-hydroxynorvaline in aphids feeding from artificial diet for 4 days (*N* = 3–5; *P < 0.05 compared to plant-fed control aphids, *t* test). (B) *S. exigua* larval weight after 9 days on artificial diet containing 5-hydroxynorvaline (*N* = 11–13). (C) Reproduction of *R. maidis* over 4 days with different concentrations of 5-hydroxynorvaline in the diet (*N* = 3–5; *P < 0.05 compared to 0mM control sample, *t* test).

5-Hydroxynorvaline was found in plant-feeding *R. maidis* and the concentration was reduced in the course of feeding for 4 days on diet without 5-hydroxynorvaline (Fig. 3A), an indication that the aphids contain 5-hydroxynorvaline that they have acquired through phloem feeding. At a concentration of 0.5mM 5-hydroxynorvaline in the artificial diet, the 5-hydroxynorvaline concentration in aphids moved from maize plants was maintained (Fig. 3A). This suggested that 5-hydroxynorvaline in the plant phloem, from which the aphids are feeding, is at a similar concentration as in the 0.5mM 5-hydroxynorvaline artificial diet.

*S. exigua* and *R. maidis* artificial diet assays were used to determine whether there is any direct toxicity from 5-hydroxynorvaline. Growth of *S. exigua* larvae on artificial diet was not significantly affected by 5-hydroxynorvaline concentrations similar to those that are present in bulk leaf tissue (*P* > 0.05, *t* test; Fig. 3B). In the case of aphids, significant deleterious effects were observed at 0.1mM 5-hydroxynorvaline and the IC_50_ (50% reduction in progeny production) was 3mM (Fig. 3C). The inhibitory concentration (0.1mM = 0.1 nmol mg^−1^) is similar to the 5-hydroxynorvaline concentration that was observed in maize leaf tissue and stems, where *R. maidis* would normally be feeding (Fig. 2H). Comparison of aphid reproduction and 5-hydroxynorvaline content in seedlings of 26 diverse maize inbred lines did not show any significant correlation (Supplementary Fig. 3), suggesting that other resistance factors may override the effects of 5-hydroxynorvaline at this growth stage.

**Fig. 4.**
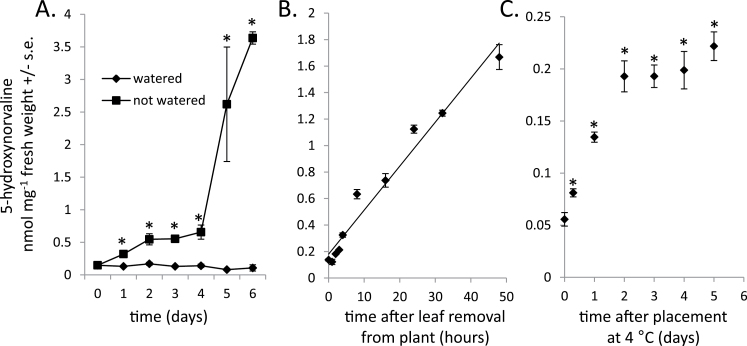
Induction of 5-hydroxynorvaline accumulation by abiotic stress. (A) Comparison of well-watered plants and plants that were not watered (*N* = 3; *P < 0.05, *t* test). (B) 5-Hydroxynorvaline accumulation in leaves that were removed from the plant and left at 23 °C. 5-Hydroxynorvaline concentration over time is expressed relative to the original wet weight of the leaves (*N* = 3; line was place by linear regression, *R* ^2^ = 0.96). (C) Concentration of 5-hydroxynorvaline in maize plants placed at 4 °C (*N* = 3; *P < 0.05, *t* test relative to 0 day or 0h controls). Means ± S.E. are shown.

As accumulation of 5-hydroxynorvaline was previously shown to be drought-induced in the foliage of other C_4_ grasses (Carmo-Silva *et al.*, 2009; Du *et al.*, 2012), similar effects were investigated in maize. In plants that were not watered there was visible wilting after 3 days and a 25-fold increase in 5-hydroxynorvaline over 6 days relative to well-watered plants (Fig. 4A), making 5-hydroxynorvaline one of the most abundant free amino acids (Supplementary Fig. 2C). Similarly, 5-hydroxynorvaline accumulated to high levels in detached leaves that were left to dry at 23 °C for 48h (Fig. 4B). The more rapid 5-hydroxynorvaline increase in detached leaves relative to intact plants may be due to a combination of faster leaf drying and wounding effects associated with leaf removal from the plants. The observed increase in detached leaves indicated that 5-hydroxynorvaline synthesis can occur in the leaves themselves and does not require transport from other plant parts, e.g. the roots. Moving plants to 4 °C, another form of abiotic stress, also increased 5-hydroxynorvaline, with a significant increase after 7h and accumulation leveling off after 2 days (Fig. 4C).

**Fig. 5.**
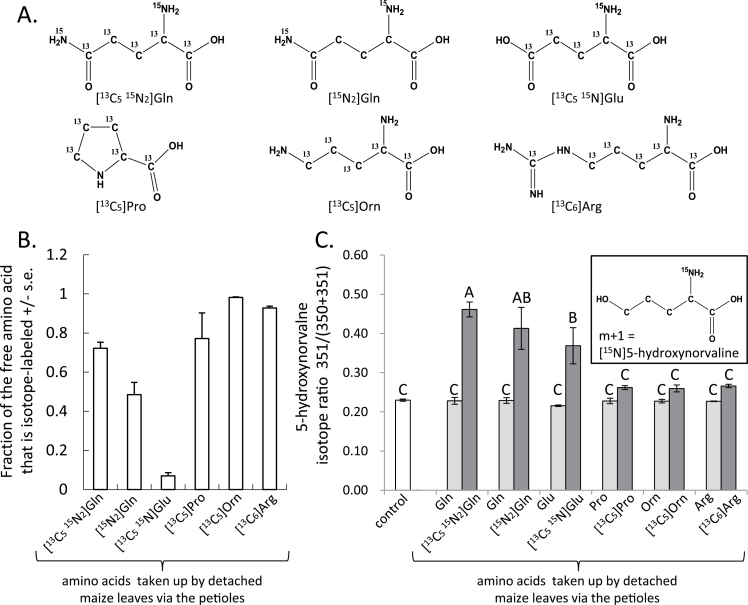
Incorporation of isotope-labelled amino acids into 5-hydroxynorvaline. (A) Structures of isotope-labelled glutamine (Gln), glutamate (Glu), proline (Pro), ornithine (Orn) and arginine (Arg) that were tested as precursors for the synthesis of 5-hydroxynorvaline. (B) Uptake of isotope-labelled amino acids via the petiole. Leaves were inserted into vials containing the indicated amino acids at 5mM concentration. Shown is the fraction of each free amino acid that is isotope-labelled. (C) Accumulation of *m*/*z* 350 (mass of trimethylsilyl-derivatized compound) and *m*/*z* 351 (m+1) 5-hydroxynorvaline (with incorporation of ^15^N, see inset) in maize leaves after uptake of unlabelled and isotope-labelled amino acid precursors via the petioles. Control samples received unlabelled amino acids. Shown is the fraction of 5-hydroxynorvaline that is m+1 (*m*/*z* 351) relative to the total 5-hydroxynorvaline in the leaves. Mean ± S.E. of *N* = 3. Different letters indicate significant differences, *P* < 0.05, ANOVA followed by Tukey’s HSD.

Stable isotope labelling experiments were used to explore the biosynthesis of 5-hydroxynorvaline in maize leaves. A possible biosynthetic pathway to 5-hdroxynorvaline biosynthesis is the reduction of γ-glutamyl semialdehyde. For instance, hydrogenase activity catalysing this reaction has been identified in *Neurospora crassa* (Yura and Vogel, 1959). Arginine, proline, glutamate, glutamine and ornithine are likely precursors for the biosynthesis of 5-hydroxynorvaline via γ-glutamyl semialdehyde, direct oxidation or other pathways (Pietzsch, 2000; Sweetlove and Moller, 2009). Therefore, detached maize leaves were placed with their petioles in solutions of [^13^C_5_^15^N_2_]glutamine, [^15^N_2_]glutamine, [^13^C_5_^15^N]glutamate, [^13^C_5_]proline, [^13^C_5_]ornithine and [^13^C_6_]arginine (Fig. 5A). GC-MS analysis showed that all of these amino acids were taken up via the maize petioles and constituted a significant portion of the free amino acid pool (Fig. 5B). However, the ^13^C backbone of these precursor amino acids was not incorporated into 5-hydroxynorvaline – that is, there was no increase in the m+5 or m+6 ions – which would indicate incorporation of the carbon backbone from the precursor metabolites. In the case of amino acids that were labelled with ^15^N (glutamine and glutamate), there was a significant increase in the m+1 ion of 5-hydroxynorvaline (*m*/*z* 351 for the trimethylsilyl-derivatized amino acid; Fig. 5C inset) relative to the background abundance, and the GC-MS fragmentation pattern was consistent with the incorporation of ^15^N. Similar amounts of m+1 5-hydroxynorvaline in experiments with [^13^C_5_^15^N_2_]glutamine and [^15^N_2_]glutamine also indicate that most of the additional mass comes from incorporation of ^15^N rather than ^13^C. Thus, although there is *de novo* synthesis of 5-hydroxynorvaline in maize, it is likely that this occurs by an as-yet-undescribed pathway.

**Fig. 6.**
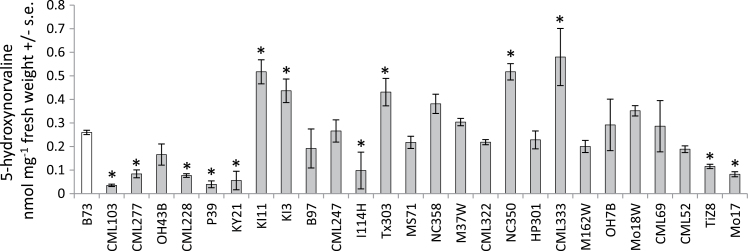
Natural variation in 5-hydroxynorvaline accumulation. Abundance of 5-hydroxynorvaline in parental lines of the maize NAM population after induction with 0.45mM methyl jasmonate. Mean ± S.E. of *N* = 3, *P < 0.05, *t* test relative to the B73 control.

To determine whether there is natural variation in accumulation of 5-hydroxynorvaline in maize, amino acid content was measured in a collection of 27 inbred lines that constitute the parents of the maize NAM population (Yu *et al.*, 2008; McMullen *et al.*, 2009). This showed a greater than 10-fold range in the accumulation of 5-hydroxynorvaline (Fig. 6), with the reference maize inbred line B73 having an intermediate phenotype. Four maize lines (CML103, CML228, CML277 and Ky21) with significantly lower methyl jasmonate-induced 5-hydroxynorvaline content than B73 were selected for mapping this quantitative trait. Joint linkage analysis of RILs derived from B73 crossed to CML103 (132 RILs), CML228 (122 RILs), CML277 (102 RILs) and Ky21 (150 RILs) identified significant QTL on chromosomes 5 and 7, which explained 16 and 20% of the total variance in 5-hydroxynorvaline content, respectively (Table 1). The 95% support intervals were from 2.1 to 2.5 Mbp on chromosome 5, containing 52 predicted genes (Supplementary Table 1) and 1.3 to 2.1 Mbp on chromosome 7, containing 42 predicted genes (Supplementary Table 2) (Maize RefGen v2; www.maizegdb.org). Composite interval mapping using individual sets of recombinant inbred lines identified significant 5-hydroxynorvaline QTL on chromosome 5 (Ky21), chromosome 7 (CML103 and CML228), and chromosome 8 (CML277) (Supplementary Fig. 4). In each case, the allele for higher 5-hydroxynorvaline content came from the B73 parent of the RIL set.

**Table 1.** Summary of joint linkage analysis of 5-hydroxynorvaline accumulation across four sets of maize recombinant inbred lines

Chromosome number	Peak position (base pairs)	95% Confidence support interval (base pairs)	Marker at the peak	*P* value at peak marker	Phenotypic variance explained
5	2183738	2136100–2501321	S_2183738	1.09×10^−10^	16%
7	1988963	1291946–2066409	S_1988963	1.01×10^−13^	20%

## Discussion

The results presented here show that accumulation of 5-hydroxynorvaline in maize leaves is induced in response to insect herbivory. Many non-protein amino acids produced by plants have defensive functions (Huang *et al.*, 2011), most commonly by substituting for other amino acids in protein synthesis or through inhibition of biosynthetic pathways. For example, *Streptococcus faecalis* ornithine transcarbamylase is inhibited by 5-hydroxynorvaline (Kurtin *et al.*, 1971). In *Escherichia coli*, 5-hydroxynorvaline competes for transport with homoserine, thereby inhibiting growth (Guirard, 1958). *In vitro* assays showed that 5-hydroxynorvaline is an inhibitor of cystathionine gamma-lyase activity (Washtien *et al.*, 1977). An as-yet-unidentified mechanism inhibits growth of *Arabidopsis* seedlings at 1mM 5-hydroxynorvaline (Heremans and Jacobs, 1994). Thus, inhibition of amino acid metabolism in *R. maidis* and/or their *Buchnera aphidicola* bacterial endosymbionts by 5-hydroxynorvaline could account for the observed reduction in their reproduction (Fig. 3C). The 5-hydroxynorvaline concentration at which aphid reproduction is reduced (0.1mM = 0.1nM mg^−1^ of diet; Fig. 3C) is similar to the concentration that is found in maize seedlings in growth chambers and less than what is observed field-grown maize plants (Fig. 2). Although there is variation in the 5-hydroxynorvaline concentration in seedlings of different maize inbred lines (Fig. 6), this was not correlated with aphid reproduction on the tested inbred lines (Supplementary Fig. 3). A likely explanation is that the variable profile of highly abundant benzoxazinoids in maize seedlings (Meihls *et al.*, 2013) may mask the possible negative effects of other plant defences. Inhibition of aphid growth by 5-hydroxynorvaline may have relevance after the seedling growth stage, when there is no longer the overriding effect of constitutive benzoxazinoids. Further research will be required to determine whether amino acid metabolism of the aphids themselves or their endosymbiont bacteria is negatively influenced by 5-hydroxynorvaline.

Drought stress induces 5-hydroxynorvaline to a higher level than insect feeding (Fig. 4). Several free amino acids, most notably proline (Verslues and Sharma, 2010), accumulate to high levels in plants in response to osmotic stress. It is thought that this amino acid accumulation promotes the survival of plant cells by acting as a compatible solute or through membrane stabilization. 5-Hydroxynorvaline could have a similar, though as-yet-uninvestigated function in maize and other C_4_ grasses that show drought-induced accumulation of this compound (Carmo-Silva *et al.*, 2009; Du *et al.*, 2012). In a previous report of drought-induced 5-hydroxynorvaline biosynthesis in C_4_ grasses it was noted that maize leaves do not contain this compound (Carmo-Silva *et al.*, 2009). Given that there is considerable natural variation in the accumulation of 5-hydroxynorvaline in maize (Fig. 6) it is possible that detection was precluded by unusually low amounts of 5-hydroxynorvaline in the maize line that was evaluated.

Stable isotope labelling experiments suggest that synthesis of 5-hydroxynorvaline does not occur via one of the predicted pathways from glutamine, glutamate, arginine, proline or ornithine (Fig. 5). However, although all of these amino acids are taken up into maize leaves, it is possible that they are not reaching the correct location for direct incorporation into 5-hydroxynorvaline. The observation that ^15^N from glutamine and glutamate is incorporated into 5-hydroxynorvaline (Fig. 5C) suggests there that there may be a maize transaminase that converts 5-hydroxy-2-oxobutanoate to 5-hydroxynorvaline, a reaction that would be similar to the final step in the biosynthesis of several other amino acids in plants. Although the incorporation of ^15^N indicates that there is at least some *de novo* synthesis of 5-hydroxynorvaline in detached leaves, it is also possible that this compound is stored as a modified precursor, for example as a glycosylated form of 5-hydroxynorvaline that is activated in response to drought.

Genetic mapping shows that at least two loci in the maize genome have significant effects on 5-hydroxynorvaline accumulation (Table 1). QTL for the accumulation of plant metabolites often co-localize with genes encoding biosynthetic enzymes. However, in the absence of further information about the 5-hydroxynorvaline biosynthesis pathway from isotope labelling studies it is difficult to identify candidate genes in the relatively large confidence intervals of these QTL. For instance, several transaminases and hydroxylases, which could contribute to 5-hydroxynorvaline biosynthesis, are located in the genetic mapping intervals. Given the variety of biotic and abiotic stresses that influence 5-hydroxynorvaline accumulation it also is possible that regulatory genes underlie the chromosome 5 and 7 QTL.

There is both complex regulation and a wide range of natural variation in the accumulation of 5-hydroxynorvaline in maize leaves, suggesting that this compound plays a significant role in responses to both biotic and abiotic stress. Most previous reports of 5-hydroxynorvaline have involved plant species for which genetic tools are not yet available (Thompson *et al.*, 1964; Brown and Fowden, 1966; Carmo-Silva *et al.*, 2009; Rohlig *et al.*, 2009; Du *et al.*, 2012). Thus, the discovery of 5-hydroxynorvaline in maize leaves provides new opportunities to investigate the biosynthesis and *in vivo* function of this plant metabolite. Fine-scale mapping of the QTL shown in Table 1, in combination with additional enzyme assays and labelling experiments, will lead to the identification of genes involved in 5-hydroxynorvaline biosynthesis. Once such genes have been identified, the isolation of knockout mutations will allow further experiments to study the *in vivo* function of 5-hydroxynorvaline in maize responses to both biotic and abiotic stresses.

## Supplementary material

Supplementary Fig. 1. MS and NMR spectra that were used for the identification of maize 5-hydroxynorvaline.

Supplementary Fig. 2. Illustration of the method used for 5-hydroxynorvaline synthesis from glutamate.

Supplementary Fig. 3. Comparison of aphid growth and 5-hydroxynorvaline content in diverse maize inbred lines.

Supplementary Fig. 4. Graphs of composite interval mapping to identify 5-hydroxynorvaline QTL in RILs derived from CML103, CML228, CML277 and Ky21 crossed to B73.

Supplementary Table 1. Predicted genes in the 5-hydroxynorvaline QTL interval on chromosome 5, from RefGen v2, www.maizegdb.org.

Supplementary Table 2. Predicted genes in the 5-hydroxynorvaline QTL interval on chromosome 7, from RefGen v2, www.maizegdb.org.

Supplementary Data

## Abbreviations:

AQC: 6-aminoquinolyl-*N*-hydroxysuccinimidyl carbamate reagent
GC-MS: gas chromatography-mass spectrometry
HPLC: high-performance liquid chromatography
NAM: nested association mapping
NMR: nuclear magnetic resonance
QTL: quantitative trait loci
RIL: recombinant inbred line.
