# Supplementary material for: Accumulation of 5-hydroxynorvaline in maize (Zea mays) leaves is induced by insect feeding and abiotic stress
Source: J Exp Bot. 2014 Sep 30;66(2):593–602. doi: 10.1093/jxb/eru385 (PMC4286406; doi:10.1093/jxb/eru385)
Supplement: Supplementary Data [file supp_66_2_593__index.html]

Accumulation of 5-hydroxynorvaline in maize (Zea mays) leaves is induced by insect feeding and abiotic stress — Accumulation of 5-hydroxynorvaline in maize (Zea mays) leaves is induced by insect feeding and abiotic stress — Supplementary Data 

# Accumulation of 5-hydroxynorvaline in maize (*Zea mays*) leaves is induced by insect feeding and abiotic stress

## Supplementary Data

Data files

**Files in this Data Supplement:**

- Supplementary Data - Supplementary Data
- Supplementary Data - Supplementary Data
- Supplementary Data - Supplementary Data
