# Supplementary material for: Accumulation of 5-hydroxynorvaline in maize (Zea mays) leaves is induced by insect feeding and abiotic stress
Source: J Exp Bot. 2014 Sep 30;66(2):593–602. doi: 10.1093/jxb/eru385 (PMC4286406; doi:10.1093/jxb/eru385)
Supplement: Supplementary Data [file supp_eru385_jexbot128835_file003.pdf]

Accumulation of 5-hydroxynorvaline in maize leaves is induced by biotic and abiotic stress

Jian Yan, Alexander E Lipka, Eric A Schmelz, Edward S Buckler, and Georg Jander

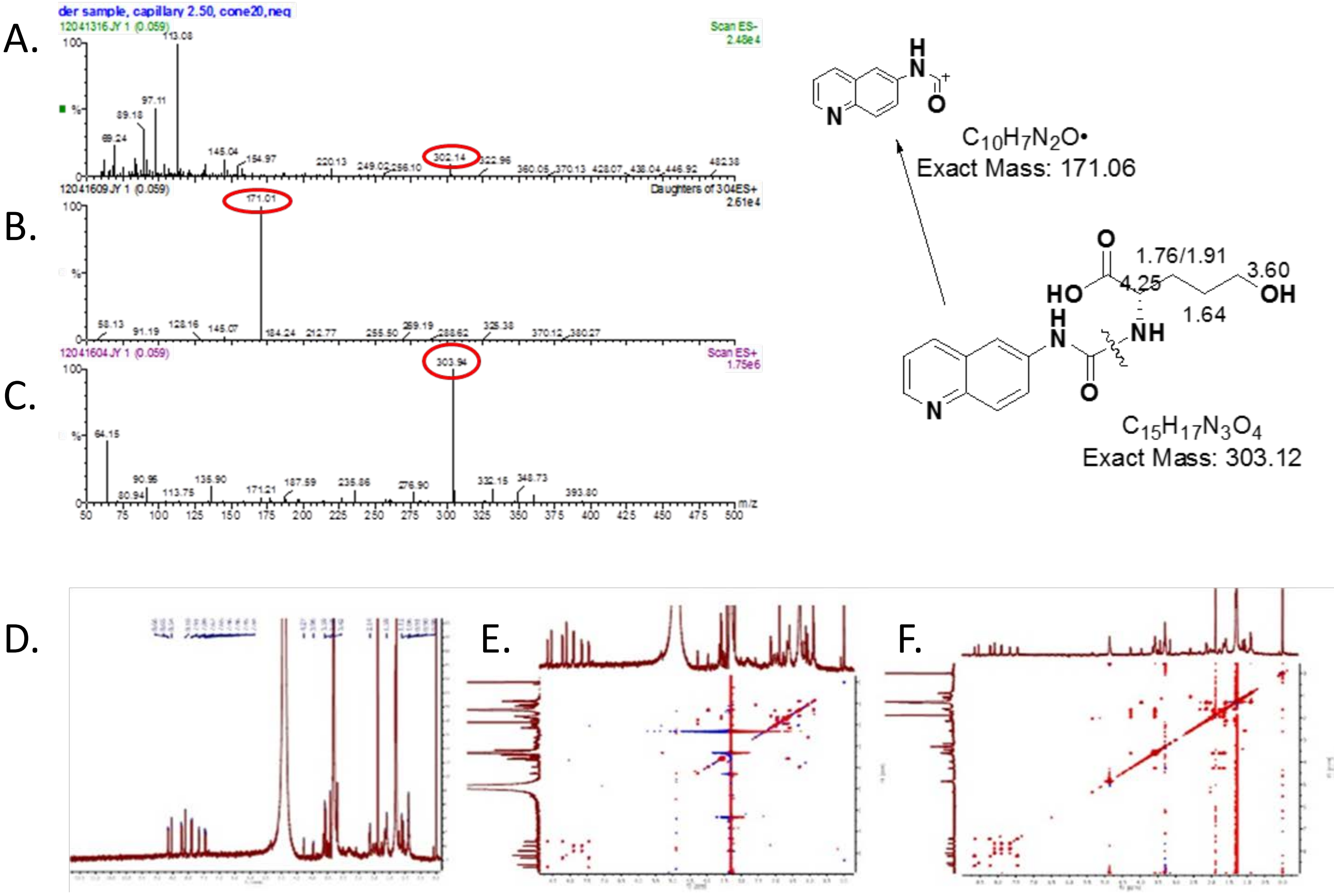

Supplementary Fig. S1. Identification of derivatized 5-hydroxynorvaline from maize tissue by HPLC-MS and NMR. (A) 5-Hydroxynorvaline negative ESI-MS spectrum, molecular weight 302.14, marked with a red oval.(B) Daughter ion peaks of 304 positive ESI-MS, key daughter ion peak 171.01, is marked with a red oval (C) 5-Hydroxynorvaline positive ESI-MS spectrum, molecular weight 303.94 marked with a red oval. (D) ^{1}H NMR spectrum, (E) 1H-1H COSY, and (F) TOCSY of 5-hydroxynorvaline.

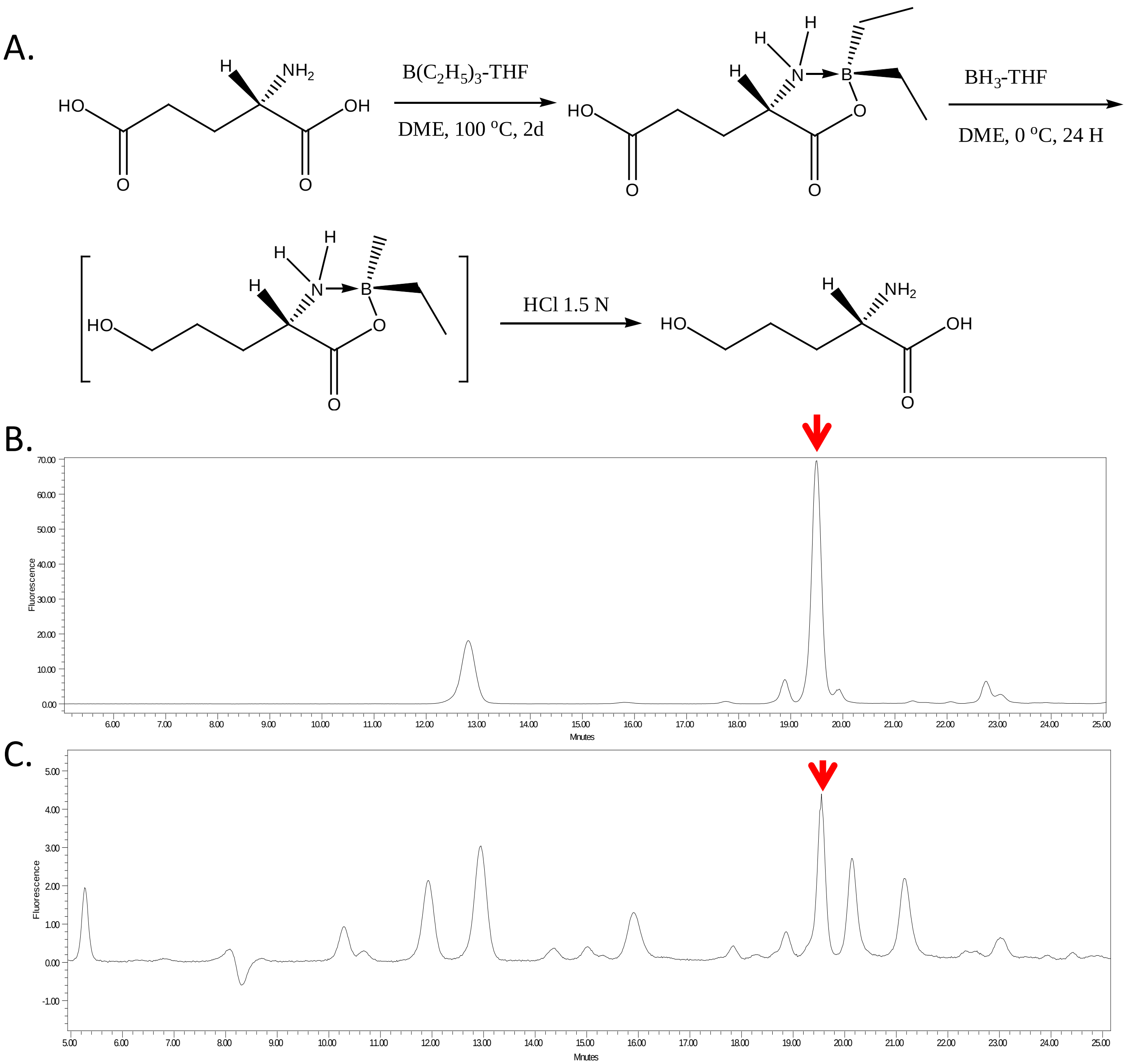

Supplementary Fig. S2. Synthesis of 5-hydroxynorvaline. (A) Synthesis by reaction of glutamate with 1,2-dimethoxyethane (DME) at 100 ºC, followed by hydrolysis with 1.5 N HCl. (B) HPLC chromatograms showing reaction products from the synthesis in panel A. (C) HPLC chromatogram of amino acids isolated from drought-induced maize leaves. In each chromatogram, 5-hydroxynorvaline peaks are indicated by arrows. For further experiments, 5-hydroxynorvaline was purified by preparative HPLC.

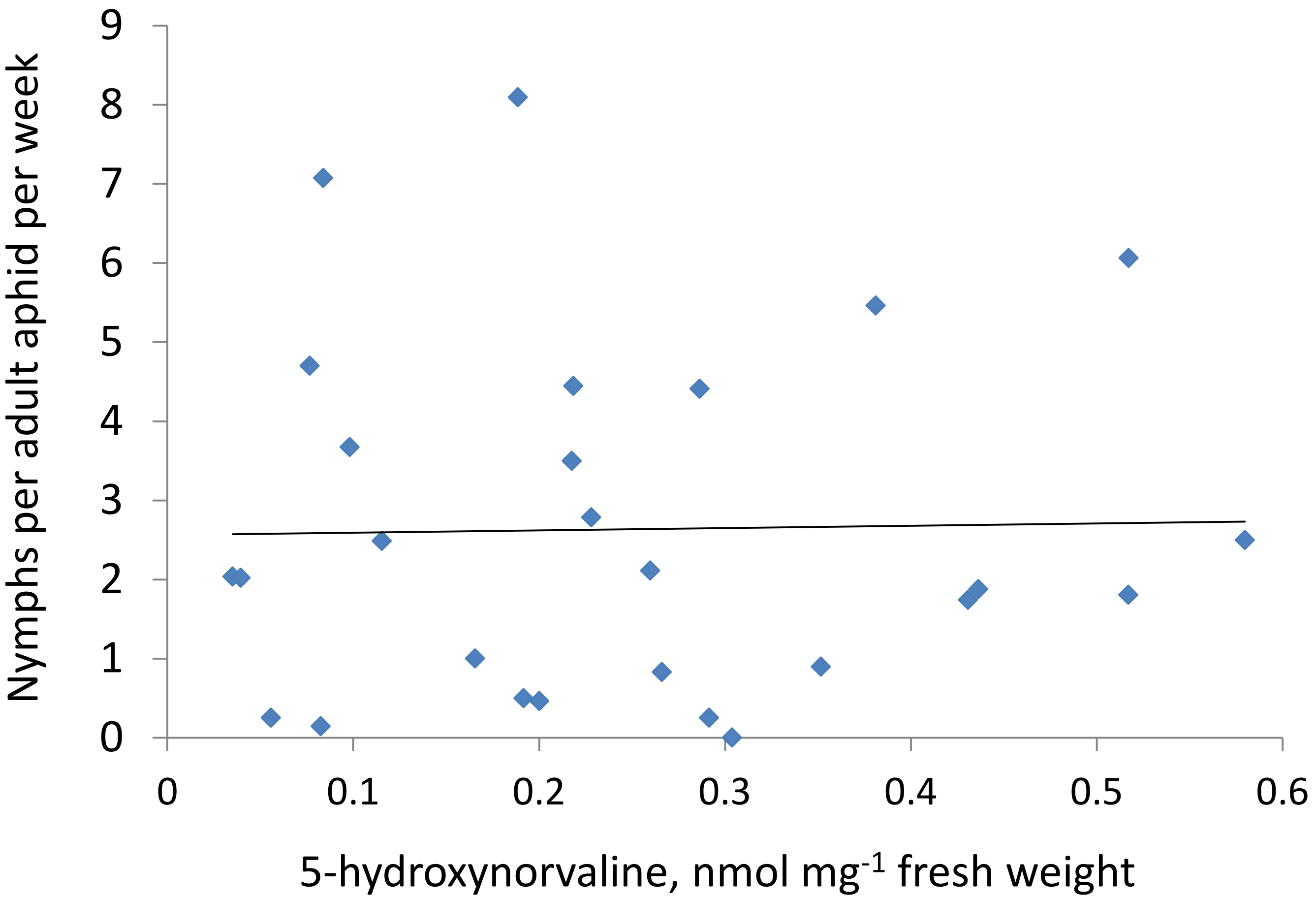

Supplementary Fig. S3. Correlation of 5-hydroxynorvaline content and aphid reproduction on NAM parental lines. 5-Hydroxynorvaline data are from Figure 6; aphid reproduction data are from Meihls et al, 2013. A best-fit line was placed by linear regression., $R^2 = 0.0004$.

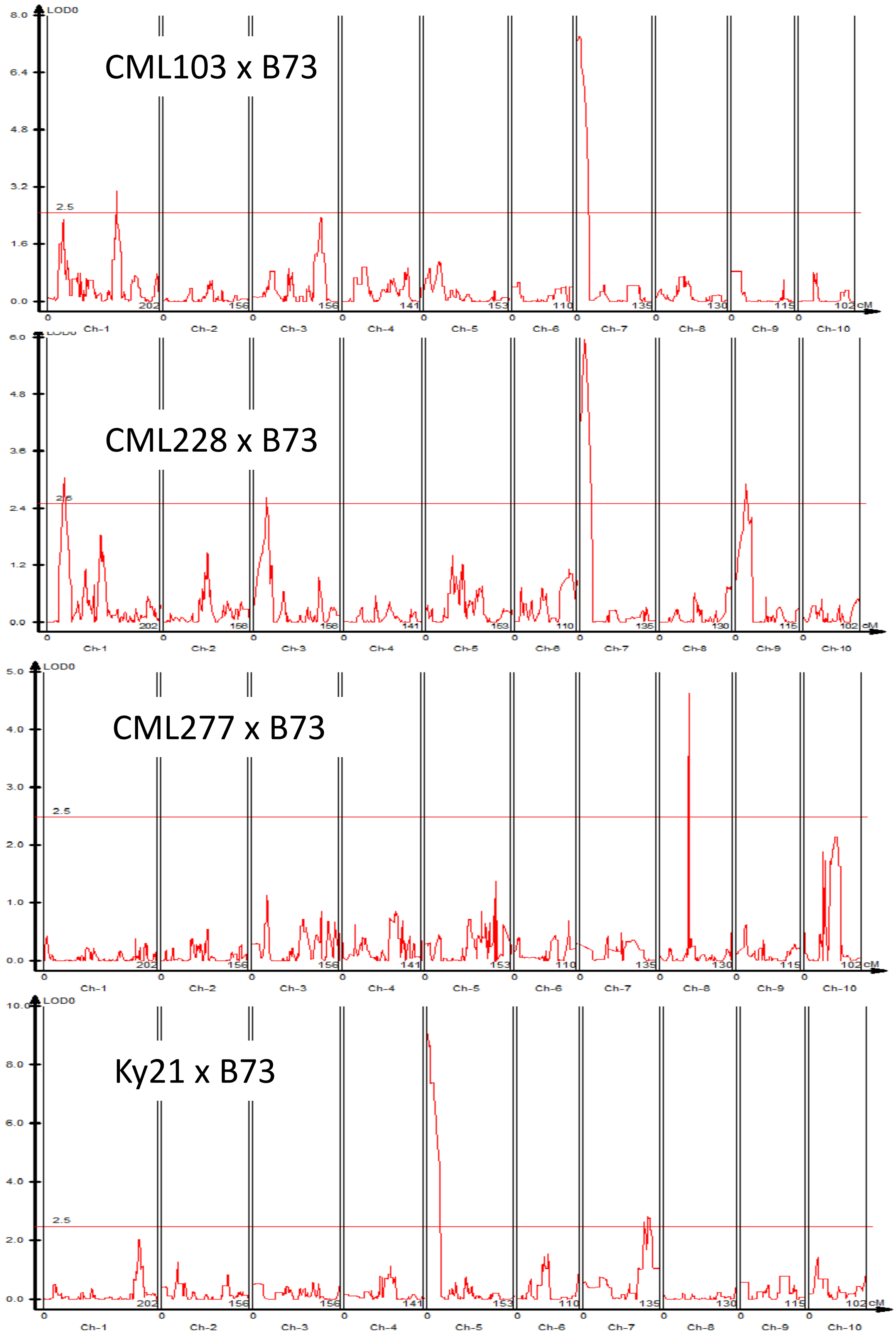

Supplementary Fig. S4. Composite interval mapping of 5-hydroxynorvaline accumulation in maize recombinant inbred lines derived from inbred lines (A) CML103, (B) CML228, (C) CML277, and (D) Ky21 crossed to B73.
